# Access to infertility care in Brazil: validation of a questionnaire for a bioethical discussion

**DOI:** 10.1186/s12978-023-01724-7

**Published:** 2023-12-08

**Authors:** Drauzio Oppenheimer, Christiane Peres Caldas, Francisca Rego, Rui Nunes

**Affiliations:** 1 Faculdade de Medicina de Itajubá (FMIT), Av. Rennó Junior, 368, São Vicente, Itajubá, MG CEP 37502-138 Brasil; 2 Faculdade de Medicina da Universidade do Porto, Porto, Portugal

**Keywords:** Infertility, Medical infertility access, Questionnaire validation

## Abstract

**Background:**

The World Health Organization characterizes infertility as a disease since 2009; however, in many countries, reproductive health is not prioritized. Characterizing a target population and knowing the barriers to accessing reproductive care may allow for a broadening of the discussion on how to provide equal opportunities. The objective of this study was to develop and validate a questionnaire that seeks to identify socioeconomic and cultural characteristics of Brazilian infertile couples to open the discussion on the principle of fair access to health.

**Methods:**

(1) literature review in the main databases, (2) questionnaire elaboration by researchers within the areas of human reproduction and bioethics, concerned with content adequacy and comprehension, using the Google Forms online platform, (3) pilot study - the questionnaire was applied to 54 couples, over 18 years of age, that were seeking treatment for infertility and accepted to participate in the study and (4) statistical analysis - for continuous numerical variables, mean, standard deviation and 95% CI of the means were presented. For discrete numerical variables, median, interquartile range, minimum and maximum were presented.

**Results:**

Forty-four questionnaires were fully completed and with adequate answers. The questionnaire proved to be objective and easy to understand. It was possible to obtain information on education, race of the couple, the impact of infertility on the couple’s life, socioeconomic conditions, and the main difficulties in accessing medical care for treatment of infertility.

**Conclusion:**

The questionnaire proved to be feasible in collecting appropriate information to characterize a target population and the only limitation was that there was no academic expert evaluation prior to the pilot test.

## Plain language summary

Although infertility has been considered a disease by the World Health Organization since 2009, there is still much disparity in access to care and in governmental policies to guarantee patients may be treated, especially in developing countries. In this study, we sought to raise awareness to this issue in Brazil, by validating a questionnaire that allows patients to express their difficulties in accessing treatment. We hope this allows for future studies that will, in time, lead to development of policies to guarantee access to reproductive care in a developing country.

## Background

The principles set forth in 1995 by the World Health Organization (WHO) in regard to reproductive health state that women have a right to physical, mental, and social welfare [1]. It is therefore imperative to provide appropriate access to reproductive care and support patients that seek to restore fertility and raise their own family [2, 3]. However, it has also been recognized that there are geographic, social, economic, political, and biological barriers that impede access to care to a large portion of men and women [4]. This leads to the need to provide better opportunities and access to reproductive medicine for patients that seek low- or high-complexity fertility treatment [5].

Many meetings – such as the 2015 American Society for Reproductive Medicine (ASRM) annual meeting, with participants from throughout the globe – have been organized to propose a strategic plan that aims at providing access to care to all infertility patients. A central point towards achieving this goal and to increase populational access to specialized care and treatments in reproductive medicine is to adequately characterize the population that faces these barriers and to understand the reasons that limit access to reproductive care [5].

Although the WHO classifies infertility as a disease since 2009 [6, 7], many specialists note that infertility—and reproductive health—is still not prioritized [8], leading to a lack of assistance or to excessive requirements to include treatments in public or private health systems [9]. Moreover, cost of treatment, age discrimination, ethnic and racial diversity, social and economic inequality, and religious and cultural beliefs further worsen conditions for these patients. Physical access is also of concern, because there is geographic concentration of practitioners and specialized assisted reproduction clinics, which further hinders patients in accessing care for infertility [5].

Although there is mobilization by medical and scientific societies and patient advocacy groups that have demonstrated disparities in access to medical treatment for infertility [5, 10], it is noteworthy that current opportunities in achieving medical assistance for maternity or paternity are largely excluding [11]. Taken together, this information demonstrates that there aren’t trustworthy data regarding current number of infertile men and women in the Brazilian population, and that these data may be skewed in the vulnerable population. Understanding this, and gathering demographic and socioeconomic data from vulnerable patients by means of a questionnaire, which allows the capture of participant feelings and opinions, may provide data so as to tailor policies and treatments in a more equanimous manner, as well as to increase ethical discussion regarding inequality in access to care.

Thus, in order to understand current limitations in access to care for a portion of the Brazilian population and to study possibilities in exercising their right to reproductive health and family planning, the objective of this study was to elaborate and validate a questionnaire that seeks to identify socioeconomic characteristics of infertile Brazilian couples and to identify how current barriers to infertility care affect vulnerable populations.

## Materials and methods

An observational study to develop a questionnaire and determine difficulties in access to reproductive care for infertile couples in the states of Minas Gerais and Sao Paulo – Brazil was carried out. An ongoing validation study is underway to broaden and update knowledge on difficulty in access to care in developing countries. A pilot study was thus conducted to validate this questionnaire. Institutional review board approval was obtained from the Itajubá School of Medicine (project number 4.920.415). Only couples seeking to achieve paternity/maternity were included.

In order to develop and validate the questionnaire, four steps were carried out: (i) literature review, (ii) questionnaire elaboration, (iii) pilot study, (iv) statistical analysis.

Literature review was carried out using the Pubmed, SciELO, LILACS, and Web of Science databases, to generate content for questionnaire elaboration. Search terms used were: access to care, assisted reproduction, biomedical ethics, fertility care, infertility and infertility questionnaire. No constraints were set for publication date or language.

The questionnaire was elaborated using Google Forms by researchers within the areas of human reproduction and bioethics, concerned with content adequacy, objectivity, clarity, ease of reading, and comprehension. Twenty-three questions were elaborated, eighteen in a nominal response scale and five open questions that aimed at identifying personal, cultural, socioeconomic identities, and the main difficulties in access to reproductive care. Questions were separated in: couple characteristics, duration of infertility, impact of infertility on couple’s life, personal experiences when seeking reproductive care and treatment, and socio-economic characteristics.

The questionnaire was applied to fifty-four couples, over 18 years of age, seeking medical treatment for conjugal infertility who consented to participate in the study. Data were collected by via Google Forms questionnaire.

It was possible to observe whether the participants found it difficult or not to answer the questionnaire and, after statistical analysis, whether the information obtained was satisfactory.

Statistical analysis was carried out using SPSS 21.0 for Windows®. Initially, a descriptive analysis of the data was performed. For continuous numerical variables, mean, standard deviation and 95% confidence interval (95% CI) of the means were presented. For discrete numerical variables, median, interquartile range, minimum and maximum were presented. For frequencies, the percentage and its 95% CI were calculated. Correlation between variables was verified using Pearson’s correlation test (between two continuous numerical variables) or Spearman’s correlation test (all other cases). An alpha error of 5% adopted for the study.

## Results

Fifty-four questionnaires were administered over a period of two months. Ten questionnaires were incomplete or with unsatisfactory answers and 44 questionnaires were fully completed and with adequate answers to the questions. Only 3 items led to no response or inconsistent answers, which were about treatment location, duration of infertility, and number of previous treatments (Table 1).

**Table 1:** Observed inconsistencies in the questionnaire

Which city will the couple perform the treatment?	*How long is the couple’s infertility?*	What were the number of attempts (treatment) to get pregnant?
14 years	homosexual couple	NO ANSWER
We haven’t chosen yet	I don’t have eggs	Infinite
	Vasectomy	Several
	undetermined	I don’t know the exact number

Age and duration of infertility data are presented in Table 2. Analysis of the partners showed that the lowest age for the wives was 24 years old and the highest age was 48 years old. In relation to the husbands, the youngest was 24 years old and the oldest was 53 years old. The time of infertility varied between 1 year and 20 years, as shown in the table below. Most wives were older than 35 years and husband age was normally distributed (p > 0.05 in a Kolmogorov Smirnov test). Most patients reported less than 10 years of duration of infertility.

**Table 2:** Age of the partners and duration of infertility

Variable	Value
Wife age (years)	
Mean (Standard Deviation)	36.5 (5.552)
95% CI	34.98; 38.02
Min – Max	24–48
Husband age (years)	
Mean (Standard Deviation)	38.83 (7.368)
95% CI	36.85; 40.84
Min – Max	24–53
Duration of infertility (years)	
Mean (Standard Deviation)	6.290 (4.102)
95% IC	5.124; 7.456
Min – Max	1–20

Demographic data are presented in Table 3. In total, 33% of wives and 29.6% of husbands had completed higher education, and all wives reported at least having completed high school education. Most wives (68.5%) and husbands (72.2%) were self-declared white. Regarding the number of previous treatments, three couples indicated numerous attempts and, therefore, were unable to inform the exact amount. Only 2 couples indicated more than 5 attempts and most of the participating couples (56%) indicated 1 or 2 attempts. A total of 13% of couples had previous paternity/maternity. Moreover, 50% of couples presented with a female cause of infertility, 14.8% a male cause, 18.5% a mixed cause, and 9 couples presented with unexplained infertility (Table 4).

**Table 3:** Demographic data

Variable	Wife	Husband
Education		
Elementary school	0	4 (7.4%)
High school	15 (27.8%)	19 (35.2%)
College degree	18 (33.3%)	16 (29.6%)
Incomplete college education	4 (7.4%)	5 (9.3%)
Post-graduation	17 (31.5%)	10 (18.5%)
None of the alternatives	0	0
Color or race		
Asian	0	2 (3.7%)
White	37 (68.5%)	39 (72.2%)
Indigenous	0	0
Brown	16 (29.6%)	11 (20.4%)
Black	1 (1.9%)	2 (3.7%)

**Table 4:** Number of previous treatments, infertility cause and previous paternity/maternity

Question. Will the couple undergo infertility treatment in another city?	
No	50%
Yes	50%
Question. What was the number of attempts (treatment) to get pregnant?	
Answer: 0	18%
Answer: 1	32%
Answer: 2	24%
Answer: 3	14%
Answer: 4	6%
Answer: 5	2%
Answer: 9	2%
Answer: 12	2%
Question. What is the cause of infertility?	
Female	50%
Male	14.8%
Mixed factor	18.5%
Infertility without apparent cause	16.7%
Question. Does the couple already have children?	
No	87%
Yes	13%

When asked about how infertility has impacted the couple’s life, 38.9% stated that infertility strengthened the couple’s relationship, 18.5% stated that the relationship had weakened and 42.6% believed that infertility did not impact the relationship either positively nor negatively. Regarding anxiety or stress due to infertility and the search for treatment, most couples (51.9%) admitted moments of anxiety from the beginning and 31.5% said they developed anxiety only after treatment attempts. Despite this, 72.2% of couples consider they have a good quality of life.

Half of the couples (50%) sought treatment in a different city from which they reside. In accordance, 42.6% of the participants reported that access to specialist and medical services was easy only after indication or assistance, and 22.2% considered it difficult to access. Additionally, even though the vast majority of participants (40.7%) rated access to an assisted reproduction clinic and treatment options as easy, 59.3% experienced difficulty or ease only after searching in another city. Even so, more than half of the couples (55.6%) did not think about giving up on treatment due to access difficulties (Table 5).

**Table 5:** Impact of infertility and difficulty accessing treatment

Question. How does infertility impact/impact the couple’s life?	
Strengthened the relationship	38.9%
Weakened the relationship	18.5%
No positively or negatively impact	42.6%
Question. Did infertility and the search for treatment cause moments of anxiety/stress in the couple’s life?	
No	16.7%
Yes, only after some treatment attempts	31.5%
Yes	51.9%
Question. The couple considers their quality of life:	
Good	72.2%
Excellent	22.2%
Bad	5.6%
Question. Access to specialist and medical services to treat infertility was:	
Difficult	22.2%
Easy	35.2%
Easy, only after indication/help	42.6%
Question. Access to the assisted reproduction clinic and infertility treatment options was:	
Difficult	20.4%
Easy	40.7%
Easy, after searching in another city/state	38.9%
Question. Has the couple ever thought about giving up on infertility treatment due to some access difficulty?	
No	55.6%
Yes	44.4%

Regarding perception of medical care, most couples (68.5%) felt welcomed when seeking medical services and treatments, and the rest of the couples were able to express whether they felt helpless or were welcomed after frustrations. Interestingly, only 26% of the couples did not seek more than one medical service, 37% sought care where they were best received and 37% sought care that was financially adequate. An important point about the search for financially adequate treatment is that 83.3% of couples were unaware of free treatment opportunities in other countries.

Furthermore, among the 5 difficulties in accessing medical services and/or treatments, financial issue (high cost of treatments) was the most selected alternative (57.4%). Location (11.1%) and discrimination (1.9%) were also recorded. Exemplifying the financial difficulty, 74.1% of couples recognized an impact on the family budget due to the cost of treatment and 55.6% needed a period between the indication of treatment and the completion of the procedure to raise financial resources. Most (61.1%) of participants did not need to resort to loans or dispose of assets to pay for treatment and 63% did not seek coverage by a health plan or the public health system for assisted reproduction treatment (Table 6).

**Table 6:** Difficulties in accessing medical services and/or treatments

Question. Did the couple feel welcomed in seeking medical services and/or treatment for infertility?	
No	9.3%
Yes	68.5%
Yes, after some frustrations	22.2%
Question. Did the couple seek more than one medical service to treat infertility?	
No, as it does not know other possibilities	13%
No, because there are not many options in your location	13%
Yes, search for adequate service financially	37%
Yes, search for the best welcome service	37%
Question. Has the couple sought or is aware of free treatment in other countries?	
No, no knowledge	83.3%
Yes, has knowledge, but has not obtained access to the other country	1.9%
Yes, has knowledge, but has not tried treatment	14.8%
Question. Point out the greatest difficulty encountered by the couple to access medical services and/or treatment for infertility:	
Discrimination	1.9%
Financial	57.4%
Location	11.1%
Location + financial	14.8%
Didn’t find difficulty access	9.3%
Other	5.6%
Question. Has the cost of treatment impacted the family budget?	
No	25.9%
Yes	74.1%
Question. Was there a need to take out loans, have assets or a similar situation to pay for infertility treatment?	
No	61.1%
Yes	38.9%
Question. Was there a need to wait between the diagnosis, the indication of treatment and the performance of the procedure to obtain financial resources?	
No	44.4%
Yes	55.6%
Question. Did the couple seek coverage from health care providers ir the public health system for treatment of infertility?	
No	63%
Yes, but they did not find any	37%

When asked about their socioeconomic status and family income, the vast majority of couples indicated tranquility, where 81.5% said they had a good socioeconomic situation, 3.7% declared an excellent situation and 14.8% thought their socioeconomic situation was bad. A family income between R$ 3,000 and 5,000 was observed in 53.7% of responders, 31.5% of the couples indicated that they had a family income between R$ 5,000 and R$ 10,000, 7.4% did not fit into the income ranges presented by the questionnaire, 3.7% answered that they had income between R$ 10,000 and R$ 15,000, and 3.7% between R$ 15,000 and R$ 20,000. No participating couple reported having a family income equal to or above R$ 25,000 (Table 7).

**Table 7:** Socioeconomic status and family income

Question. The couple considers their socioeconomic status:	
Good	81.5%
Excellent	3.7%
Bad	14.8%
Question. What is the gross family income?	
R$ 3.000 A R$ 5.000	53.7%
R$ 5.000 A R$ 10.000	31.5%
R$ 10.000 A R$ 15.000	3.7%
R$ 15.000 A R$ 20.000	3.7%
R$ 25.000 OR MORE	0%
None of the alternatives	7.4%

Additionally, in order to know the range of the population that seeks treatment for infertility, the questionnaire showed that three out of four couples (75.9%) know friends or have acquaintances who have sought treatment, and several participating couples (37.5%) pointed out that these friends were not able to access infertility treatments.

Correlation analysis showed that 95.7% of patients who sought assistance at another city than where they live had already attempted at least one treatment cycle, while only 68% of patients who sought treatment where they lived had already attempted at least one treatment cycle (p = 0.024).

It is important to note that, in accordance with results presented above, 61.5% of couples who sought treatment in another city/state found it easy to access the assisted reproduction clinic and treatment options only after seeking treatment in a different city from which they reside. Also, 57.7% of couples that did not seek treatment in another city, considered they had easy access (p = 0.003).

The questionnaire also reported that 42.3% of couples who did not seek treatment in another city sought more than one medical service to treat infertility. Furthermore, 46.2% of couples who sought treatment in another city were looking for a more receptive environment (p = 0.032). 73.1% of couples who responded they had not sought treatment in another city and 92.3% who had reported they were not aware of free treatment in other countries. However, expectedly, 26.9% of couples who did not find treatment in another location admitted to having knowledge about the gratuitousness in other countries (p = 0.048).

Finally, it was possible, by this questionnaire, to obtain information about the main difficulties in accessing medical care to treat infertility, where 80.8% of couples who did not seek treatment in another city pointed out that the high cost is the greatest adversity for carrying out the treatment. In accordance, 30.8% of respondents who selected to have sought treatment in another city also found the financial issue as the main limiting factor. Furthermore, it was shown that 23.1% of the couples in the same group indicated the location, that is, living in a city with few resources, as the greatest difficulty encountered by the couple to achieve treatment (p = 0.003).

## Discussion

Infertility is a worldwide public health problem [9], but access to care is heterogeneous, which makes it necessary to observe populations in a more individualized way in order to obtain perspective on impacts of infertility in men and women under diverse location, financial, and social realities. An instrument was thus developed and validated to address these aspects within a population of infertile couples. It is important to note that the development of the questionnaire took into account validation of the research instrument, so that there is methodological coherence and consistency of results [12, 13].

The questionnaire developed sought to identify the socioeconomic positions of several patients with infertility who sought medical assistance in São Paulo and Minas Gerais (Brazil), and to identify difficulties in access to reproductive care experienced by the target population, in order to better understand the limits of access to reproductive care and, with that, plan and develop mechanisms to exercise the right to reproductive health and family planning. In this validation study, we observed that the questionnaire was simple to administer, and feedback from patients showed that it was easy to understand, with no need for adjustments. In addition, results demonstrated internal consistency, so that the questionnaire is a valid instrument and allows obtaining information that is statistically possible to be analyzed.

Results analysis allowed us to characterize the main characteristics of participants, indicating age, duration of infertility, level of education, and color or race. Furthermore, it made it possible to understand the patients’ anxiety with the open answers regarding number of treatment attempts, where some couples indicated that they no longer keep track of the count, and referring to the biggest difficulty encountered by the couple to access medical services and/or treatment, where couples were able to express their particular difficulty. Additionally, the last two items of the questionnaire showed how there is a still unknown infertile population.

Corroborating with several authors [5] and as an important factor in discussions aimed at reproductive health rights, the main barriers to reaching treatment were cost of treatment, followed by concentration of clinics in larger cities. Financial sensibility was also an important deciding factor, and a driving force in leading couples to seek treatment alternatives. In addition, the questionnaire exposed how couples assess their quality of life, focusing on the socioeconomic status and possible stress and anxiety in the couple’s life. Finally, correlation analysis brought data that support the information acquired, managing to exemplify the main difficulties in accessing treatments to treat infertility and the search for its scope.

In conclusion, the questionnaire proved to be feasible in collecting appropriate information to characterize a target population; that is, the questionnaire was able to meet the main purpose of the study, and the only limitation found was that there was no evaluation by academic judges prior to the pilot test. Even so, the questionnaire proved to be easy for couples in the survey to understand.

## Data Availability

Not applicable.
